# Overuse of artemisinin-combination therapy in Mto wa Mbu (*river of mosquitoes*), an area misinterpreted as high endemic for malaria

**DOI:** 10.1186/1475-2875-7-232

**Published:** 2008-11-05

**Authors:** Charles Mwanziva, Seif Shekalaghe, Arnold Ndaro, Bianca Mengerink, Simon Megiroo, Frank Mosha, Robert Sauerwein, Chris Drakeley, Roly Gosling, Teun Bousema

**Affiliations:** 1 Department of Medical Microbiology, Radboud University Nijmegen Medical Centre, Nijmegen, the Netherlands; 2 Kilimanjaro Christian Medical Centre, Moshi, Tanzania; 3 Kilimanjaro Christian Medical College, Moshi, Tanzania; 4 KKKT Kirurumo Health Facility Mto wa Mbu, Tanzania; 5 Joint Malaria Programme, Moshi, Tanzania; 6 Department of Infectious and Tropical Diseases, London School of Hygiene and Tropical Medicine, London, UK

## Abstract

**Background:**

Adequate malaria diagnosis and treatment remain major difficulties in rural sub-Saharan Africa. These issues deserve renewed attention in the light of first-line treatment with expensive artemisinin-combination therapy (ACT) and changing patterns of transmission intensity. This study describes diagnostic and treatment practices in Mto wa Mbu, an area that used to be hyperendemic for malaria, but where no recent assessments of transmission intensity have been conducted.

**Methods:**

Retrospective and prospective data were collected from the two major village health clinics. The diagnosis in prospectively collected data was confirmed by microscopy. The level of transmission intensity was determined by entomological assessment and by estimating sero-conversion rates using anti-malarial antibody responses.

**Results:**

Malaria transmission intensity by serological assessment was equivalent to < 1 infectious bites per person per year. Despite low transmission intensity, > 40% of outpatients attending the clinics in 2006–2007 were diagnosed with malaria. Prospective data demonstrated a very high overdiagnosis of malaria. Microscopy was unreliable with < 1% of slides regarded as malaria parasite-positive by clinic microscopists being confirmed by trained research microscopists. In addition, many 'slide negatives' received anti-malarial treatment. As a result, 99.6% (248/249) of the individuals who were treated with ACT were in fact free of malaria parasites.

**Conclusion:**

Transmission intensity has dropped considerably in the area of Mto wa Mbu. Despite this, most fevers are still regarded and treated as malaria, thereby ignoring true causes of febrile illness and over-prescribing ACT. The discrepancy between the perceived and actual level of transmission intensity may be present in many areas in sub-Saharan Africa and calls for greater efforts in defining levels of transmission on a local scale to help rational drug-prescribing behaviour.

## Background

Adequate diagnosis and prompt treatment of malaria remain major difficulties in rural settings in sub-Saharan Africa. In these areas, more than 70% of individuals with symptoms suggestive of malaria treat fevers with anti-malarial drugs without visiting the formal health sector for diagnosis [1,2]. If people do visit a health facility, an accurate diagnosis is not guaranteed. Microscopic examination of a blood smear is the gold standard method for the diagnosis of malaria, but is often unavailable at sub-district facilities [3]. In the absence of microscopy, malaria diagnosis is based on clinical symptoms that are known to lack specificity [4], or treatment is administered presumptively. Even if microscopy is available, there is substantial overdiagnosis of malaria [5]. There is an urgent need to review these diagnostic and treatment practices with the wide-scale implementation of the relatively expensive artemisinin-combination therapy (ACT) and in the light of changing patterns of malaria transmission.

Presumptive treatment of malaria remains a useful tool for malaria control in areas where malaria is common, the risk of progression to severe malaria is significant, and diagnostic facilities are lacking. In these areas, presumptive treatment is an effective strategy to increase the coverage of anti-malarials and may act to reduce transmission of malaria [6]. However, the strategy may not be justifiable for low endemic areas where the majority of febrile episodes are not due to malaria [7] and over-diagnosis and over-treatment of malaria are very common [4,8,9]. Knowledge of malaria transmission intensity can guide clinicians in defining algorithms for dealing with febrile patients. However, reliable estimates of malaria transmission intensity are frequently unavailable and transmission intensity can change over time as a result of interventions [10,11] or natural fluctuations [12]. Across the African continent, there are reports of recent reductions in malaria transmission intensity (reviewed in [6]).

Investigating how rural health systems function in the current climate of changing transmission and efforts to control and eliminate malaria seems pertinent. Here, diagnostic and treatment practices in rural health centres in Mto wa Mbu are described, an area that was historically hyperendemic for malaria [13], but where no recent assessments of transmission intensity have been conducted.

## Methods

### Study site

This study was conducted in Mto wa Mbu (latitude 3° 21'S; longitude 35° 51' E), which is translated from Kiswahili as 'River of Mosquitoes'. The town is located in the northern highlands of Tanzania at an altitude of 960 m – 1478 m above sea level and rain fall is largely restricted to the short (Oct-Nov) and long rainy season (March-July). According to a national census that was conducted in 2002, the town has 16,068 inhabitants. Retrospective and prospective data were collected from the two major village health clinics: the clinic of the Evangelical Lutheran Church (KKKT) and the Roman Catholic Health Facility (RCHF).

### Retrospective data

Both clinics routinely used microscopy for diagnosing malaria and reported malaria to be their primary diagnosis in outpatients. Retrospective data consisted of information from outpatient registers of the years 2006 and 2007. For each individual that presented at the clinic the visit date, age and sex was recorded, as well as if they were diagnosed with malaria (either by clinical assessment or after detection of parasites by the clinic microscopist) and whether anti-malarial treatment was given. Climatic data were obtained from the nearby Lake Manyara National Park.

### Prospective data

Data was prospectively gathered from all individuals attending the clinics as patients in the months May-August 2007, at the end of the long rains when a high number of malaria cases was expected [12,14]. All patients were informed about the purpose of the study and asked to give written consent before inclusion in the study. Once enrolled, a brief questionnaire was administered to collect information on: age, sex, bed net use, use of antibiotics or anti-malarials in the previous two weeks and symptoms. A single finger prick blood sample was taken for serum collection on Whatman 3 MM filter paper [Whatman, Maidstone, UK]. If individuals were referred for malaria diagnosis, a finger prick blood sample was taken for malaria parasite detection by Rapid Diagnostic Test (RDT) and two blood slides. ParaHIT^® ^RDTs [Span Diagnostics Ltd, Surat, India]. RDTs were used according to the manufacturer's instructions and were based on the detection of Histidine Rich Protein 2. The first of the two blood slides was stained at the clinic according to the clinic's routine practice and scored by the microscopist working at the clinic. This slide result and the RDT result were made available to the clinician for diagnosis. The second slide was later stained in a research lab and read by two experienced research-microscopists. Parasite density per 200 white blood cells was determined on the thick smear and the slide was considered negative if no parasite was observed in 100 microscopic fields. Results of the two research microscopists were compared for validation and all discordant were read by a third microscopist.

Individuals who were diagnosed with malaria by the clinician received artemether-lumefantrine in line with the Tanzanian national policy. This study received ethical clearance from the ethical board of the Kilimanjaro Christian Medical Centre (KCMC Ethical Clearance certificate 2007 #167).

### Entomology

Mosquitoes were collected in 10 houses that were selected to be representative of the different housing structures in the village. Mosquitoes were caught with a standard Centre for Disease Control light traps (CDC, Atlanta, GA, USA) every fortnight for three months (May – September 2007). Traps were hung at the end of an occupied bed with an untreated bed net that was newly provided by the investigators. Traps were set for 12 hours, from 7 pm to 7 am [15,16]. In the morning, traps were collected and mosquito species determined and counted. Male *Anopheles *mosquitoes, Culicines and non-vector Anophelines were discarded. Female *Anopheles *mosquitoes were stored on silica gel for circumsporozoite protein (CSP) ELISA as described by Wirtz *et al *[17]. The head and thorax were removed for every mosquito, prepared for ELISA [17] and stored in an uncoated 96-well microtitre plate until the assays were performed. Samples were prepared individually and assayed with positive mosquitoes repeated. Insectary reared unfed female Anophelines were used as negative controls together with a commercially provided CSP positive control [CDC, Atlanta, USA]. Samples were read by eye and on an ELISA plate reader at 495 nm. The EIR and confidence intervals were calculated as described by Drakeley *et al. *[18]. A conversion factor was used to adjust for the difference between light trap catches and man biting catches, giving the formula: infectious bites per person per month = 1.605 * (number of positive mosquitoes/number of traps) * 30 [18].

### MSP-1 ELISA

Serum was eluted from filter papers as described by Corran *et al *[19]. Immunoglobulin G antibodies against blood stage antigens were detected by indirect ELISA, as previously described [20] using recombinant MSP-1_19 _(Wellcome genotype), which were produced as described previously [21]. Briefly, flat bottom 96-well plates [Immulon 4HBX, Thermo] were coated overnight with 50 μL of 0.5 mg/mL dilution of the specific antigen. After washing with PBS-0.05% Tween [(PBS-T), 200 μL of blocking buffer (1% skimmed milk in PBS-T) was added for 3 hours at room temperature. After washing, plasma samples were added in duplicate at a single dilution of 1/1000 and incubated at 4°C overnight. 100 μL of rabbit anti-human IgG HRP Conjugate [Dako, Ely, UK] was added and incubated for 1 hour at room temperature. Plates were developed with o-phenyline-diamine [Sigma]-H_2_O_2 _and the reaction was stopped with 50 μL H_2_SO_4_. Plates were read at 490 nm. To generate an OD cut-off value above which samples were deemed antibody positive, the distribution of OD values was fitted as the sum of two Gaussian distributions (assuming a narrow distribution of seronegatives and a broader distribution of seropositives) using maximum likelihood methods [19].

### Data analysis

Statistical analyses of data were performed using SPSS version 14.0 and Stata 9.2 (Stata Corp, College Station TX, USA). Categorical variables were compared between groups by the Pearson Chi-square test or Fisher's Exact test, odds ratios (OR) with 95% confidence intervals (95% CI) were calculated. MSP-1_19 _ELISA data were used to generate an age-seroprevalence plot. OD values were expressed as percentage of the positive control (normalised OD) The annual seroconversion rate, λ, and the annual rate of reversion to seronegativity, ρ, were estimated by fitting a simple model of the acquisition and loss of antibodies to the age-specific prevalence of the antibodies using maximum likelihood methods assuming a binomial distribution [20]. The equivalent annual entomological inoculation rate (EIR) was then estimated using a calibration curve derived from previously determined values [22].

## Results

### The perceived burden of malaria

**Figure 1:**
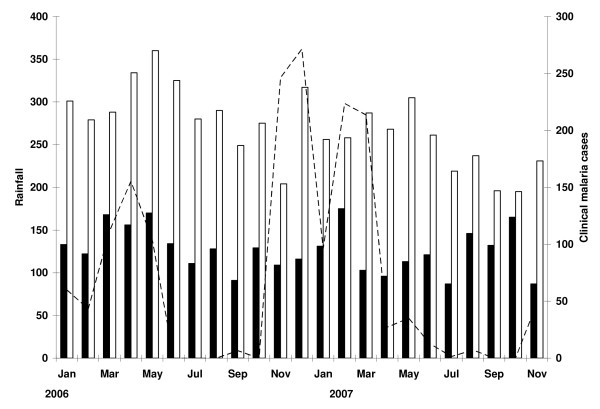
**Rainfall distribution and number of outpatient visits diagnosed with malaria**. Bars indicate the number of clinically diagnosed malaria patients younger than five years of age (closed bars) or ≥ 5 years of age (open bars) in the KKKT and RCHF clinics combined in the period January 2006 – November 2007. The dashed line indicates the total monthly rainfall in mm/month measured at the nearby Lake Manyara National Park.

In the period January 2006 – November 2007, the two clinics registered a total of 22,553 outpatient visits. In this period, 62.9% (1696/2696) of the outpatient visits of children below five years of age were diagnosed with and treated for malaria at the KKKT clinic, compared to 39.2% (3284/8382) in individuals ≥ 5 years. In the RCHF, these figures were 42.9% (1914/4466) and 42.1% (2948/7009), respectively. There was no evident seasonal pattern in the total number of malaria cases while clear peaks in rainfall were recorded in the months of March-June and October-December (Figure 1). Total rainfall was 650 mm per annum.

### The measured burden of malaria

#### Malaria case management and microscopically confirmed parasitaemia

**Figure 2:**
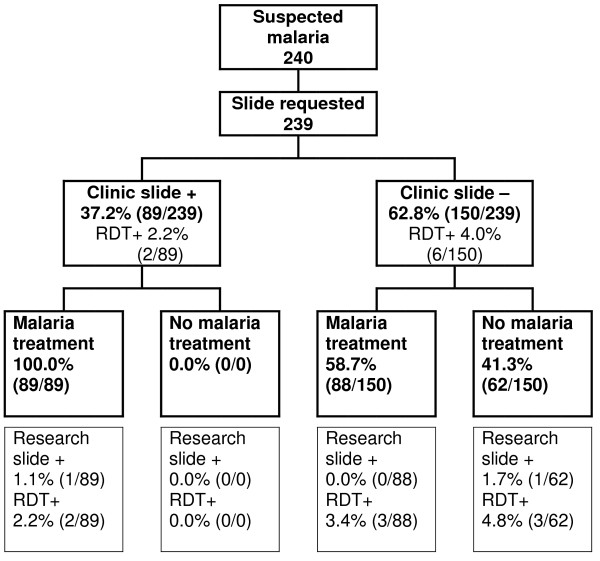
**Management of individuals with suspected malaria at the KKKT clinic**. Clinic slide = slide stained and read by clinic microscopist; research slide = slide stained and read by trained research microscopist; RDT = rapid diagnostic test

**Figure 3:**
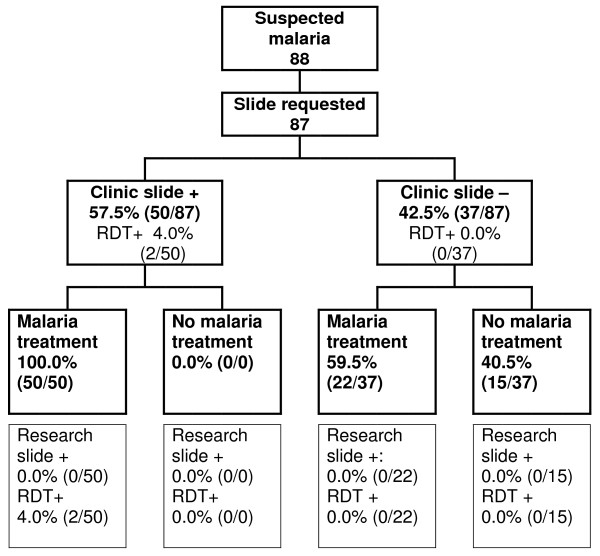
**Management of individuals with suspected malaria the RCHF clinic**. Clinic slide = slide stained and read by clinic microscopist; research slide = slide stained and read by trained research microscopist; RDT = rapid diagnostic test

The clinical management of malaria cases was observed in the period June – August 2007 at both clinics. In the KKKT clinic, 240 individuals attended the clinic with symptoms that the clinician interpreted as suggestive of malaria (Figure 2); in the RC clinic this number was 88 (Figure 3). Reported complaints, age and socio-demographic factors were similar between the two clinics. The most commonly reported complaint was fever (80.9%, 263/325) followed by respiratory complaints (41.8%, 136/325). The median age of suspected malaria cases was 26.5 years (IQR 10.0 – 38.0) and 51.5% (168/326) reported the use of anti-malarials in the two weeks preceding their visit. Bed net use was reported by 85.1% (417/490) of the individuals without information on (recent) impregnation.

Slide and RDT results and the prescribed anti-malarial treatment were summarized in Figures 2 and 3. A slide was requested for almost all individuals with suspected malaria (99.3% (326/328); the remaining two received anti-malarials without laboratory testing. All individuals who had parasites on their blood slide according to the clinic microscopist received anti-malarial treatment, both at the KKKT clinic and the RC clinic. Of those who were considered parasite free by the clinic microscopist, 58.7% (88/150) and 59.5% (22/37), nevertheless received anti-malarials at the KKKT and RC clinic, respectively. At the KKKT clinic 37.2% (89/239) of all requested slides were scored positive by the microscopist but only one of these 'positive slides' was confirmed by the research microscopists. The proportion of 'positive slides' was even higher in the RC clinic where 57.5% (50/87) of all requested slides was considered positive by the clinic microscopist and none of these were positive when stained and read by the research microscopists. One parasite positive slide was missed by the microscopist in the KKKT clinic (parasite density: 27,760 parasites/μL) and was also RDT negative; this person received antibiotics, but no anti-malarials. There was no statistically significant association between slide positivity by the clinic microscopists and the research microscopists (p = 0.84). Ten individuals were positive by RDT, one of them was also slide positive and two others reported the previous use of anti-malarials.

When data of the two clinics were combined, the following factors were associated to prescription of anti-malarial drugs: the chance of receiving anti-malarials was increased when the slide was scored positive by the clinic microscopist (OR 1.70; 95% CI 1.51–1.92) and decreased in case of reported cough (OR 0.50 95% CI 0.30 – 0.84). Reported fever and reported previous use of anti-malarials or antibiotics were not associated with the chance of receiving anti-malarials at the clinic.

#### The entomological inoculation rate by mosquito sampling

A total of 70 CDC light traps were set in the period May-September 2007. Of the mosquitoes caught, 15.6% (331/2774) were of the *Anopheles *genus and 261 were female. All female mosquitoes were processed and included in the CSP ELISA, one was CS positive. This resulted in an estimated entomological inoculation rate (EIR) of 0.69 (95% CI 0.02 – 3.83) infectious bites per person per month for the period surveyed.

#### The entomological inoculation rate equivalent generated from seroprevalence data

**Figure 4:**
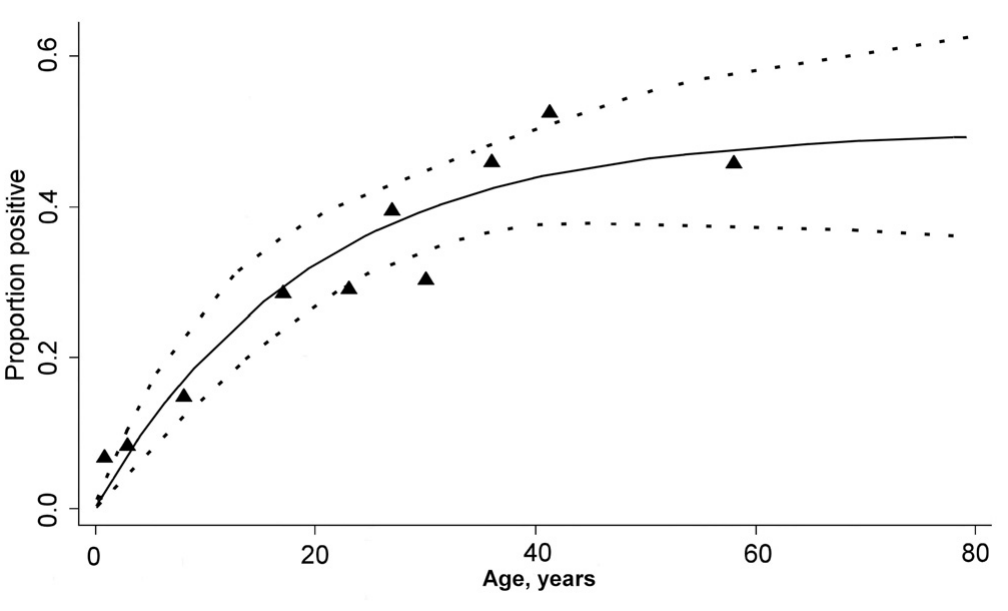
**Age specific seroprevalence plot of anti-MSP-1_19 _antibodies**. The vertical axis shows the proportion of seropositive individuals in each age group, the horizontal axis shows the midpoint age of each age group. Filled triangles represent the raw data; the lines represent the fitted equation with parameters λ = 0.026 and ρ = 0.026 and the 95% confidence interval.

A total of 464 serum samples were tested in the MSP1_19 _ELISA. The overall seroprevalence of MSP1_19 _antibodies was 29.5% (137/464) and there was a clear increase in seroprevalence with age (Figure 4). Based on the fitted curve, the λ was estimated at 0.026 (95% CI 0.016 – 0.042), which corresponds to an estimated EIR of 0.70 (0.26 – 1.87) infectious bites per person per year.

## Discussion

In the study area of Mto wa Mbu that is characterised by low transmission intensity, an unrealistically high perceived burden of malaria was observed. Forty percent of outpatients were treated for malaria while this diagnosis was not supported by blood slide or rapid test and was improbable in the light of the actual level of transmission intensity.

The level of malaria transmission intensity, as determined by rapid entomological assessment and MSP-1_19 _age-seroprevalence data [20], was low in the area of Mto wa Mbu. The EIR estimate by entomological assessment was 0.7 infectious bites per person per month. This estimate was obtained in the period after the long rains and probably represents the peak exposure to infected mosquitoes. It is, therefore, not surprising that it exceeds the EIR estimate based on the MSP1_19 _age-seroprevalence curve (0.7 infectious bites per person per year) although the confidence intervals for both methods overlap. In general, EIR by entomological assessments is susceptible to seasonal fluctuations [12], fluctuations that are smoothened out by the immunologic assessment [20,22] that may, therefore, give a more robust estimate. Either method will lead to the conclusion that transmission intensity in Mto wa Mbu is low. This is in contrast with cross-sectional surveys from 1981 [13], when parasitological surveys indicated that the area was having a level of transmission intensity 'certainly equal to and probably higher than that found in Muheza-Ubembe' [13], an area that is still known to be hyperendemic for malaria [23]. Malaria transmission intensity clearly decreased in last 25 years but the period in which the reduction took place is unclear. (Recent) reductions in transmission intensity can be detected in age seroprevalence curves when the force of infection (λ) is allowed to change over time [22]. However, a variable λ did not improve the fit of our curve (data not shown), and we found no indications for a recent reduction in EIR. Similar to its timing, the reasons for the reduction in EIR are unclear. The use of chloroquinized salt can not explain this since it was used in the 1960s and 70s and parasite rates of 50–60% in children < 10 years of age were still reported in 1981 [13]. Bed net coverage was high in the study area, but the coverage with ITNs has only increased in recent years [24] and also provides no plausible explanation. Similar to other areas in Africa [6], the drop in transmission intensity is not easily explainable. The high use of anti-malarials for any fever treatment, as demonstrated in this study, could have acted as mass prophylaxis and reduced transmission over time [6], but this cannot be proven.

Despite the low transmission intensity in the area of Mto wa Mbu, more than forty percent of all outpatients who attended two major clinics over a period of almost two years were diagnosed with malaria. There was no seasonal pattern in malaria diagnoses, as is commonly observed [12,14]. Prospective data from more than 400 suspected malaria cases demonstrate that there was a massive overdiagnosis of malaria in the two clinics. Despite the availability of microscopes, experienced microscopists and clinicians, who frequently requested slides, the targeting of anti-malarials appeared to be unimproved [5,25]. One major explanation for this was that slide readings were unreliable in the clinics included in this study: less than 1% of slides that were regarded as malaria parasite-positive by clinic microscopists were confirmed by trained research microscopists. In addition, clinicians appeared to use blood slide results more as a tool to confirm their clinical suspicion rather than to rule out malaria [25]. All individuals with 'positive slides' received anti-malarial treatment although a 'negative slide' did by no means rule out treatment [9]. This could be a result of the deeply entrenched belief in slide negative malaria. It is true that clinical malaria can result from low density parasitaemia in low endemic areas and malaria can therefore not always be ruled out in slide-negative cases [4]. However, these are exceptional cases and restricting anti-malarials to true microscopy-positives is a safe approach, even in areas of low endemicity [8]. The prescription of anti-malarials in Mto wa Mbu was clearly out of proportion. An astounding 99.6% (248/249) of the individuals who were treated with artemether-lumefantrine (AL) was free of malaria parasites.

Although a recently published hypothesis suggests that overdiagnosis and overtreatment of malaria can have a beneficial prophylactic effect in malaria control [6], the current findings are worrying for several reasons. Firstly, our area is of low endemicity and a beneficial prophylactic effect is unlikely [6]. Secondly, diagnosis of malaria and according treatment may simply be a 'convenient' clinical strategy avoiding the more complicated search for other causes of the presenting illness [25]. Treatment of all febrile episodes as malaria is likely to result in underdiagnosis of other fever-causing disorders such as childhood pneumonia [2]. Thirdly, over-treatment occurred with the expensive AL. AL and other ACTs are typically 10-times more expensive then previously used drugs as sulphadoxine-pyrimethamine [26,27] making reliable diagnosis crucial for cost-effective use [28]. The artemisinin component in ACT also do not have the prophylactic effect that was suggested to be beneficial in 'opportunistic presumptive treatment' [6]. Artemisinin is eliminated from the circulation in a matter of hours [29] leaving the partner drug, in this case lumefantrine, unprotected. That leads us to the fourth reason for unease. There is concern for a reduced susceptibility of *P. falciparum *parasites for ACT [29] and the spread of parasites with reduced susceptibility to ACT may be enhanced by irrational drug use [30]. Reports on allelic selection after artemether-lumefantrine [31] provide additional warnings against over-use of ACT.

For a way forward, it is important to understand why there is so much overdiagnosis and overtreatment in the study area. A recent study elsewhere in Tanzania has demonstrated that patient pressure, traditionally mentioned as a major contributor [32], may not be important in overtreatment [33]. Patients often prefer to be slide-tested and treated in line with results [33]. In the clinics in Mto wa Mbu, slide reading was clearly inadequate. Microscopists will need additional training and in addition, more objective diagnostic tools such as RDTs can play a role in the improvement of diagnosis. Clinicians in this study felt uncomfortable to rule out malaria based on a negative RDT (and one patient in our study was in fact a false RDT negative). Time will be needed to make them consider RDTs as useful diagnostic tools [34]. Perhaps delivering a message to health workers and the public explaining that malaria control has been successful in some areas and malaria has truly been reduced may improve the situation. If both health staff and public understand that this fever may not be due to malaria quality of care may improve and "routine overdiagnosis" may be a story of the past.

## Conclusion

The observed discrepancy between the perceived and actual level of transmission intensity may be present in many areas in sub-Saharan Africa and calls for greater efforts in defining levels of transmission on a local scale. National policies may have to give way to more sensible local policies that use proven multiple interventions in areas of high-moderate transmission and focus on accurate diagnosis and treatment in low transmission settings.

## Competing interests

The authors declare that they have no competing interests.

## Authors' contributions

CM, AN, BM and SM were responsible for data collection and were involved in data analysis and manuscript preparation; SS and HM were involved in statistical data analysis and manuscript preparation; FM and RS in study design; CD, RG and TB were responsible for study design, data interpretation and manuscript preparation.
